# Rhythm Patterns Interaction - Synchronization Behavior for Human-Robot Joint Action

**DOI:** 10.1371/journal.pone.0095195

**Published:** 2014-04-21

**Authors:** Alexander Mörtl, Tamara Lorenz, Sandra Hirche

**Affiliations:** 1 Institute for Information-Oriented Control, Technische Universität München, Munich, Germany; 2 Experimental Psychology, Ludwig-Maximilians-Universität, Munich, Germany; University of Sheffield, United Kingdom

## Abstract

Interactive behavior among humans is governed by the dynamics of movement synchronization in a variety of repetitive tasks. This requires the interaction partners to perform for example rhythmic limb swinging or even goal-directed arm movements. Inspired by that essential feature of human interaction, we present a novel concept and design methodology to synthesize goal-directed synchronization behavior for robotic agents in repetitive joint action tasks. The agents’ tasks are described by closed movement trajectories and interpreted as limit cycles, for which instantaneous phase variables are derived based on oscillator theory. Events segmenting the trajectories into multiple primitives are introduced as anchoring points for enhanced synchronization modes. Utilizing both continuous phases and discrete events in a unifying view, we design a continuous dynamical process synchronizing the derived modes. Inverse to the derivation of phases, we also address the generation of goal-directed movements from the behavioral dynamics. The developed concept is implemented to an anthropomorphic robot. For evaluation of the concept an experiment is designed and conducted in which the robot performs a prototypical pick-and-place task jointly with human partners. The effectiveness of the designed behavior is successfully evidenced by objective measures of phase and event synchronization. Feedback gathered from the participants of our exploratory study suggests a subjectively pleasant sense of interaction created by the interactive behavior. The results highlight potential applications of the synchronization concept both in motor coordination among robotic agents and in enhanced social interaction between humanoid agents and humans.

## Introduction

Synchronization is frequently observed across different modalities and situations. In particular, the synchronization of movements is found to play an essential role in the interactive behavior of humans. Due to its ubiquity in human life, interpersonal synchronization is experimentally investigated in various tasks that require jointly performed movements in a shared workspace: When walking in a group, humans tend to synchronize their gait [1]. Two people sitting next to each other in rocking chairs are found to synchronize their rocking movements [2], even if the natural frequencies of the chairs differ. Similar behavior is observed in laboratory tasks such as pendulum swinging [3] or pure leg movements [4]. Even during goal-directed tapping that requires precise arm movements [5], synchronization among human dyads is emerging naturally without being instructed or demanded for the task. In this task, interpersonal movement synchronization can be clearly quantified as a coupled dynamical process [6]. Studies on the social aspects of synchronization highlight that falling into synchrony with partners enhances perceptual sensitivity toward each other, fosters cooperative abilities [7] and leads to the attribution of more positive characteristics to the interaction partner [8]. These works give rise to the hypothesis, that bidirectional motor coordination with synchronization as its key concept is a promising way to increase the social competence of robots when interacting with humans [9].

Inspired by the appealing prospect to enrich the interaction repertoire of robots, this article addresses the challenge of designing interactive behavior for artificial agents engaging in repetitive *joint action tasks*. These tasks involve actions performed by two or more individuals in a common social setting, inducing action coordination in space and time [10]. Based on synchronization theory of coupled dynamical systems [11], we present a synchronization concept for repetitive, goal-directed movements composed by mixed continuous and discrete primitives.

### Movement Synchronization among Humans and Machines

One line of research on human synchronization behavior follows the dynamical systems approach. Patterns of coordination are considered to result from attractors of dynamical systems, that model interconnected perception-action loops [12]. This concept is also called behavioral dynamics [13]. Investigating intrapersonal limb coordination, Haken et al. [14] propose a minimal dynamical model of coupled oscillators, which is known as the Haken-Kelso-Bunz (HKB) model. It reproduces the main coordination features observed during rhythmic bi-manual finger-tapping. The HKB model family qualitatively explains interpersonal movement synchronization in rhythmic paradigms as well [2], [15]. In this vein, the rigorous design of rhythmic movement behavior by dynamical systems in the state space is performed by Jirsa and Kelso [16], which the authors call the excitator model. Common to these approaches is the monolithic encoding of movement coordination and reproduction, making them rather task specific. A second line of research on human synchronization behavior is devoted to the intended synchronization of human rhythmic movements with respect to purely discrete, periodic stimuli such as auditory metronome beats, which is often called sensorimotor synchronization (SMS). Linear models of asynchrony correction based on the Wing-Kristofferson model [17] explain perceptual and motor variabilities from an information-processing point of view, see [18], [19] for an exhaustive review. It is recently debated whether hybrid incarnations of both the dynamical systems approach and the linear error correction concept may exist [20] or not [21], or whether these model classes simply account for different synchronization processes present in the task [22]. Irrespective of the underlying process, it is found that discrete perceptual information such as distinguishable events during continuous movements provides anchoring points for time keeping with a stimulus and, thus, fosters human SMS [23]. Notably, humans are found to rapidly adjust their pacing toward each other during dyadic finger tapping, thus improving coordination by mutually coupled SMS [24].

Limit cycle systems creating rhythmic movement based on self-sustained oscillators are also called central pattern generators (CPGs) in robotics. Entrainment tasks, such as robot drumming [25], are modeled by CPGs, where phase locking regarding the beats is achieved. An extension of the CPG approach by reconfigurable dynamical systems is proposed by Degallier et al. [26] to generate mixed discrete and rhythmic movements in multiple degrees of freedom. The encoding of periodic movements based on adaptive frequency oscillators is realized in Gams et al. [27] and developed further by Petric et al. [28]. Frequency and phase tuning shows a rather slow rate of convergence for non-stationary trajectories. Though CPGs model robust and flexible motor behavior, an open issue is the missing methodology to systematically design and specify CPGs in a task-oriented way. For profound reviews on the design and application of CPGs, the reader is referred to [29], [30]. Some works investigate human-machine rhythmic coordination. Mutual entrainment of movements is achieved by rendering visual or acoustic stimuli to the human as real-time feedback. The concept of virtual partner interaction (VPI) is introduced in [31]. In a proof-of-concept implementation, the coordination of finger movements between a human and a visually-rendered, virtual agent driven by the HKB model is explored systematically. In various applications, rhythmic entrainment between humans and robots is investigated. Popular examples are human-robot rope turning [32], [33] or the imitation of human rhythmic movements of selected target frequencies [34] by means of phase-locked loops (PLL) [35]. Both human-robot handshaking [36] and physical assistance for rhythmic knee movements [37] are realized based on the Matsuoka neural oscillator [38]. Here, Sato et al. [39] achieve encoding of rhythmic movements and implicit synchronization through an on-line polynomial design of the attractor dynamics, which is originally proposed by Okada et al. [40].

However, the above works focus either on fundamental research of human synchronization behavior or, within human-machine interaction, on applications in purely rhythmic tasks. To the authors’ best knowledge, none of the existing works, except our previous [5], [6], analyzes and models synchronization of hybrid action tasks composed by mixed continuous and discrete primitives with application to human-robot dyads.

### Contribution

In this article, we develop a concept of movement synchronization for repetitive joint action tasks. Those tasks are assumed to be described by closed movement trajectories that can be goal-directed and comprise multiple primitives. The modeling concept pursued in our analytical work on human synchronization behavior [6] is generalized to enable the design of synchronization behavior for robotic agents in a wide range of tasks: Based on oscillator theory, limit cycle representations of the trajectories in state space are used to derive the phase variable, even for sequences of multiple primitives. Relevant synchronization modes within pairs of limit cycles are synthesized considering both continuous phases and discrete events from a unifying point of view. In line with the behavioral dynamics perspective [13], [41], we design a dynamical process to synchronize the derived modes. Movement generation is addressed as well, in order to enable a robotic agent that is equipped with synchronization behavior engage in repetitive joint action tasks. The presented experimental study employing a full-sized, anthropomorphic robot serves not only as proof of concept; it also defines a versatile testbed for the investigation of human-robot interactive behavior in realistic settings.

The remainder of this article is organized as follows. First, we clarify the required assumptions and definitions. Based on those, synchronization modes are analyzed and dynamical synchronization behavior is designed accordingly. Next, the required transformations between phase variables and movement trajectories are developed. After the design concept, we describe the human-robot experiment, its implementation and the applied measures. A detailed assessment and evaluation of the implemented synchronization behavior is presented. After a discussion of the results and insights, we sum up and draw the conclusions.

Bold characters are used for denoting vectors in this article. Superscripts ^a^ and ^b^ are used when variables belonging to agent ‘a’ and agent ‘b’ need to be distinguished. For clarity these superscripts are omitted otherwise.

## Problem Setting and Definitions

This section provides the reader with the formal representations and definitions that are used in this article to characterize the joint action task as well as movement synchronization.

### Representation of Repetitive Joint Action Tasks

The notion of joint action [10], originated from cognitive psychology, is adopted in this work, whereas we extend joint action to robotic agents as well. Let each agent’s part of the joint action task, which we call the individual task, be represented by a state trajectory 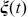, i.e. the evolution of the vector of relevant states 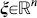 over time *t*. The state vector can be composed by the configuration of the agent’s limbs, their hand (effector) position, or any other coordinates that describe the movements associated with the individual task.

*Note.* A certain set of states is considered suitable if the information conveyed through the chosen description allows to explain and model the synchronization behavior of the agents.

#### Limit cycle trajectory

**Figure 1:**
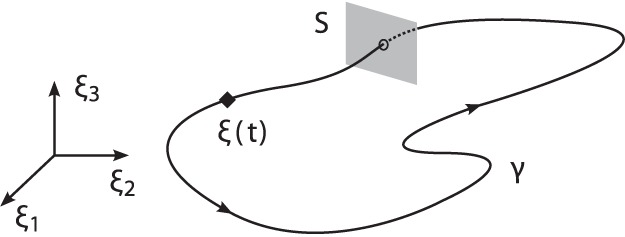
Limit cycle 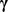 of an exemplary cyclic state trajectory 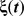 in its state space with 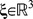. If 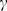 is cyclic, yet not closed exactly, the period 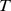 is determined by the return time of 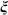 to the Poincaré secant surface 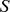.

*Note.* For trajectories obtained from noisy measurements, condition (1) is relaxed by examining the return times to the Poincaré secant surface [42], allowing for 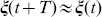.

The concept of movement synchronization exploits the repetitive aspect of the individual task. Therefore, the state trajectory is required to be *cyclic*, i.e. for any time *t* and finite time spans 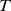 the condition
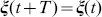
(1)holds. The smallest 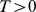 which fulfills (1) is denoted the *period*. It follows that the state space representation of 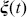 is of circular shape, which is denoted the *limit cycle* 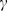, see Fig. 1. Due to interaction between the agents, the period 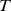 is time-varying and consequently, 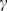 is strictly speaking not periodic.

#### Primitives, events and durations

**Figure 2:**
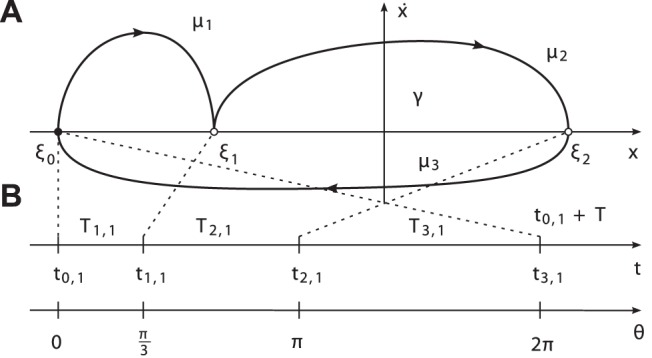
Characterization of the limit cycle. (A) Exemplary limit cycle 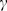 with the state 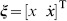 and 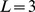 primitives. The segmentation points 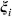 are given by the intersection of 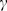 with the abscissa. (B) The corresponding events 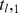, primitive durations 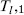 and the uniformly growing phase 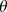 depicted for 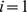.

*Note.* The segmentation points are assumed to be such that any task-related constraints on the state can be satisfied, e.g. goal points or forbidden state regions.

The limit cycle 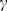 is assumed to be composed by a number of 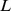 segments 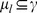 in an ordered sequence 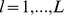. These are called *primitives*. Each primitive 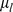 is delimited by two segmentation points, the start point 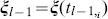 and the end point 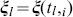, as illustrated in Fig. 2(a). The positive index 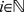 denotes the 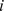
th period. The period is taken by 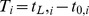 in the following. It has to be noted that 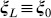 and 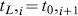 respectively, since 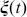 is cyclic. The times 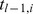 and 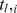 are called *events*, see Fig. 2(b). Without loss of generality, we choose segmentation points featuring discriminable events, such as local extrema of the movement with vanishing velocity [43]. Segmentation points with zero or negligible velocity persisting for non-zero time intervals are considered as postures [44] and separate dwell primitives respectively. Those dwell primitives are also delimited by event pairs, denoting the times of movement stop and start. Discriminable events in cyclic trajectories are shown to support human mechanisms of temporal error correction [23], and thus affect human synchronization behavior.

The times 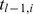 and 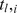 define the *primitive duration*

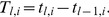
(2)


Relating the *current* primitive duration 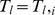 and the *current* period 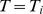 with index 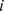 such that 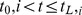, we further define the *relative primitive duration*

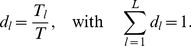
(3)


The distribution 
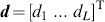
 gathering 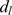 in a vector scales the primitive durations 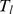 under modulations of 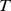.

### Synchronization of Limit Cycle Pairs

The limit cycle 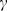 is assumed to be originated from a self-sustained oscillation, which allows us to apply the theory of limit cycle oscillators. The notion of phase is introduced to describe the motion of the state on the limit cycle. The definition of synchronization relates both the phase and events of a pair of limit cycles to each other and therefore, characterizes coordination in time.

#### The phase variable

Through a coordinate transformation, the limit cycle is re-parameterized by the one-dimensional variable 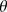 that is called the *phase* and describes the motion on 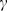 and the (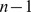)-dimensional vector of *amplitudes* that describe motions transverse to 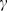. This transformation is not unique, and thus, different decompositions can be found for a certain limit cycle [45]. In our setting of goal-directed tasks, we assume the amplitudes to be constrained by the segmentation points delimiting the primitives, compactly gathered in 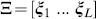. Consequently, only the phase is considered to be governed by synchronization in the following.

Among all possible transformations, we choose the phase obtained from the harmonic phase oscillator, which is one of the simplest oscillator models. Its unperturbed oscillations evolve at constant phase velocity 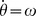, with 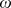 denoting the natural frequency. Accordingly, its phase trajectory is defined
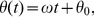
(4)which is growing uniformly in time. By setting 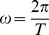, we further define the phase to be angular and 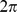-periodic, evaluating
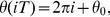
(5)with any initial phase 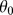. Finally, the phase 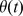 needs to be uniquely related to the state 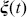. We deliberately choose 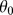 such that 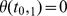 is anchored to the point 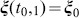 marking the event 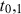, cf. Fig. 2(b). The phase of a stationary limit cycle with constant period 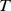 is readily given by (4), which is analogous to the marker technique in [42]. The important case of a non-stationary limit-cycle with a-priori unknown period 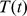 is addressed later.

*Note.* The above transformation can be understood as a decomposition of the task into the phase, which is the *voluntary* degree of freedom available for synchronization, and the amplitudes, which are the remaining degrees of freedom *necessarily* complying with the task goals.

#### Phase and event synchronization

With the above definition of phase and under the assumption that the phase is originated from a self-sustained oscillating entity, a synchronization problem between a pair of phase oscillators is posed accounting for a dyad’s coordination in time. The oscillators are assumed to be mutually coupled through some coupling function and completely described by their phases 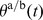 defined on the limit cycles 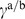. If the phase difference
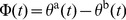
(6)is bounded by a positive constant 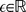


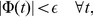
(7)the limit cycles show *phase synchronization* [11] of order 1∶1. Higher order synchronization is not addressed in this article for the sake of simplicity.

In addition, the quasi-simultaneous appearance of event pairs is considered, known as event synchronization [46]. Let 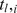 denote the time of the 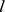
th event in the 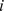
th period of 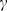. We define, that the event pair denoted by the tuple 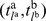 shows *event synchronization*, if the events keep the temporal relation
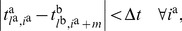
(8)with some time span 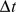. Choosing 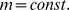, with 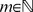 ensures to test for event synchronization of order 1∶1. The choice of 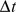 is considered as problem dependent. To avoid ambiguities, a reasonable upper bound is given by
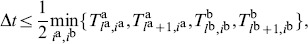
(9)which is half the minimum primitive duration or half the minimum inter-event distance in the neighborhood of the considered pair 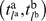.

*Note.* The above notion of event synchronization implies phase synchronization, since the time lag and thus, the phase difference between the considered events is bounded. Event synchronization depending on the definition of relevant events provides a problem-specific characterization of the temporal organization of two limit cycles.

## Design of Synchronization Behavior

Following the above definitions of phase and event synchronization and inspired by principles of human movement synchronization, in this Section we design synchronization behavior with application to repetitive joint action tasks. Accounting for the derived descriptions of possible synchronization modes, a unified synchronization process is developed.

### Synchronization Modes

After analyzing the common modes of the synchronization between quasi-harmonic trajectories, which usually result from rhythmic movement tasks, we broaden the repertoire of potential synchronization modes between limit cycles featuring multiple primitives and events.

#### Modes between harmonic limit cycles

**Figure 3:**
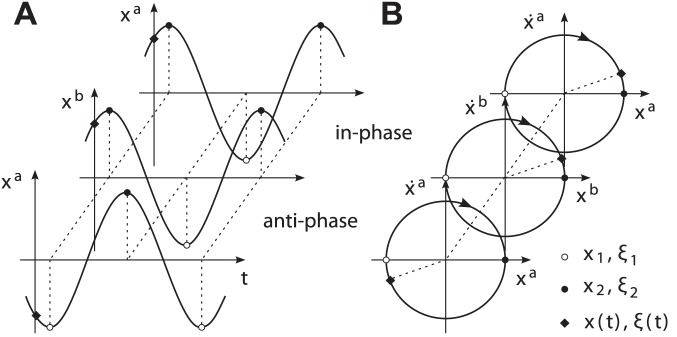
Modes between harmonic oscillations. Phase synchronization resulting in in-phase or anti-phase relations comes about with event synchronization with respect to the segmentation points 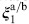 and 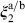. (A) Motion trajectories 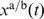 describing the temporal relation. (B) Their limit cycle representations 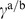 in a position-velocity state space, illustrating the phase difference.

Research on movement synchronization within human dyads has mainly focused on task paradigms requiring purely rhythmic movements such as finger tapping, leg or pendulum swinging. These tasks are usually described by one-dimensional motion trajectories, e.g. with the state 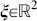 embedded in a position-velocity state space. Typically, each period of the trajectory is composed by two nearly equal and sinusoidal half-periods, allowing to treat the oscillation as harmonic. Following the definitions made above, the limit cycle 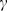 of the state trajectory 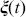 is segmented into 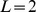 primitives 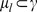, 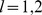, which are symmetric due to their relative primitive durations with 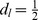 being constant and equal, cf. Fig. 3. For pairs of limit cycles 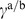 originated from harmonic oscillations, the notions of the *in-phase* and the *anti-phase* relation usually characterize the common modes of synchronization. When we calculate the relative phase difference
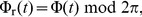
(10)with 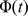 from (6) and mod denoting the mathematical modulo division, the in-phase and the anti-phase mode map to 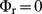 and 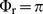 respectively, cf. Fig. 3(b).

These modes are equivalently described by event synchronization according to the above definition of synchronization. Evaluating the phase (4) at the event 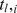 yields with (3)
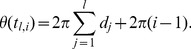
(11)


Thus, we obtain 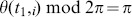 and 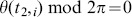 for symmetric primitives with 
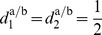
. It follows that the relative phase difference (10) evaluates 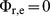 and 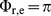, if the event pairs 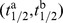 and 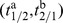 appear synchronized. Summing up, quasi-harmonic cycles are considered to be composed by two symmetric primitives and events respectively. Their common synchronization modes are sufficiently described by the phase dynamics of coupled oscillator models, e.g. [6], [14], [47], [48].

#### Modes between multiple-primitive limit cycles

**Figure 4:**
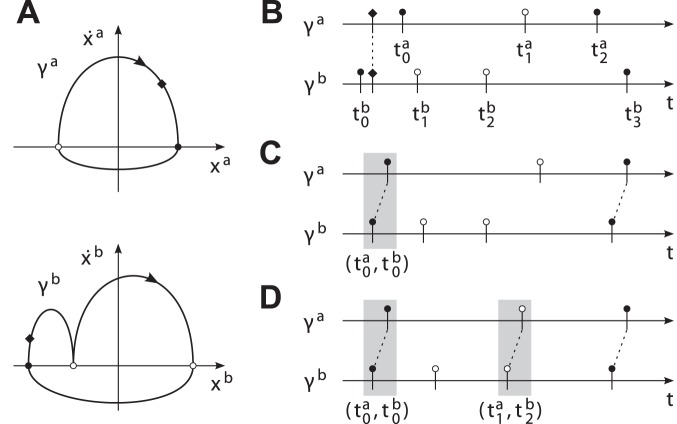
Event synchronization of heterogeneous limit cycle pairs. (A) Exemplary limit cycles 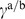 with 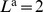 and 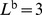 primitives in position-velocity state spaces. The evolution of the events in 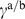, (B) without synchronization, (C) with synchronization of the event pair 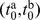 as achieved by phase synchronization, (D) with additional synchronization of 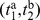. The shaded areas denote the time span 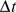 defining event synchronization.

In repetitive joint action tasks, the limit cycles 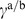 represent the agents’ individual tasks. Those can be composed by different sequences of multiple primitives, i.e. with the number of primitives 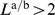, the distributions of relative primitive durations 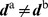, or both. Here, the relevant modes of synchronization are assumed to describe the (simultaneous) synchronization of one or more event pairs 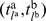, see the modes in Fig. 4(c)–(d).

The example in Fig. 4 illustrates, that phase synchronization is not sufficient to describe all of these modes. Phase synchronization models stable equilibrium points 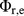 of the phase difference which lead to 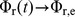 and imply 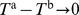 in the domains of attraction. This allows to synchronize single event pairs, like the one depicted in Fig. 4(c). If the within-cycle distributions of events differ 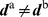 like in our example, the simultaneous synchronization of not more than one event pair is explained by the phase dynamics, since the events scale under changes of 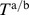 with the distributions 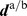, which are, however, left uncontrolled. Obviously, the simultaneous synchronization of *multiple* event pairs requires an additional adjustment of 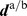, see Fig. 4(d).

*Note.* Only a task-dependent subset of events might be synchronized, e.g. only those that are perceived by the interaction partner.

### Dynamical Synchronization Process

Synchronization behavior is modeled in line with the *dynamical systems approach* [13], which explains stable behavioral patterns by attractors of dynamical systems. First, we review the phase dynamics modeling the synchronization of human dyads performing quasi-harmonic limit cycles in a goal-directed movement task. The above analysis shows, that phase synchronization is able to account only for a limited number of possible synchronization modes. Therefore, we design a unified synchronization process that features the simultaneous synchronization of multiple event pairs.

#### Model of coupled phase oscillators

In accordance to the definition of phase synchronization, the model structure is given by a pair of cross-coupled phase oscillators
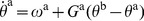
(12)




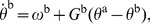
(13)with the natural frequencies 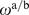, and the coupling functions 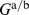 depending on the phase difference between the oscillators. By subtracting (13) from (12), we obtain the phase difference dynamics
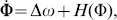
(14)with 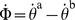 and the frequency detuning



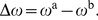
(15)The function 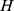 is the vector field of 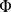 forming the attractor landscape, and thus, the preferred modes of phase synchronization.

*Note.* Synchronization behavior is assumed to be voluntary and compliant with the task-related goals. We therefore require the coupling functions to be weak and 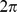-periodic, i.e. equilibrium points 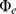 are equivalently described by equilibrium points 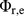 of the relative phase difference (10) between the oscillators. Consequently, a large enough frequency detuning 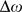 completely eliminates stable attractors, which is found to be in line with unintentional coordination behavior of humans [49].

**Figure 5:**
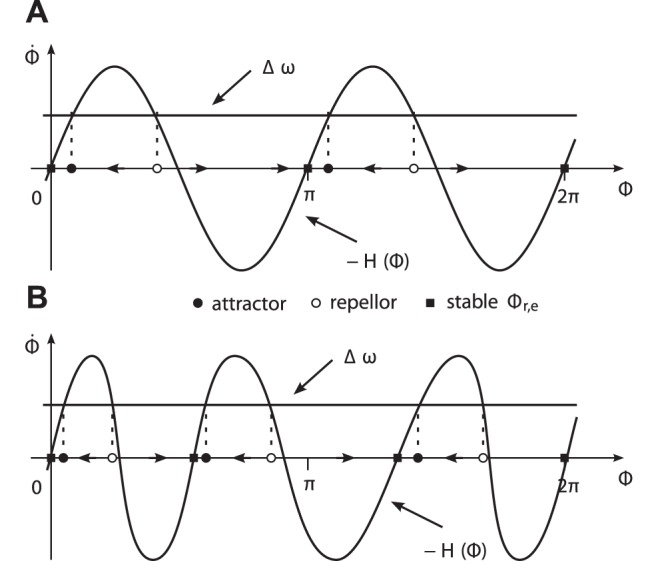
R.h.s. terms of the phase difference dynamics (14) over Φ∈[0,2*π*]. The intersection points of the graphs of 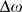 and 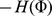 denote equilibria with 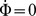. The vector fields are illustrated on the abscissae. (A) The extended Kuramoto model featuring two equally-spaced attractors. (B) Exemplary phase dynamics featuring three attractors determined via (19).

*Note.* The extended Kuramoto model implies equal attractor strengths, as both attractors were met nearly equally often in the experimental task.

In the following, we review a realization of the coupling functions 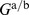 that accounts for the observed process of inter-human movement synchronization [6]. The proposed model structure is based on the classical *Kuramoto model* [50]. Its equations of motion read
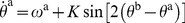
(16)




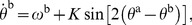
(17)which we call the *extended Kuramoto model*. The natural frequencies model the individually preferred speed of task performance, whereas the sinusoidal coupling with the isotropic gain 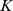 replicates the dyad’s interactive behavior. We obtain the phase difference dynamics
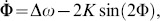
(18)featuring two point attractors around 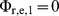 and 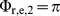, see Fig. 5(a) for a visualization of the vector field. The dynamics (18) replicate the synchronization process of human dyads that leads to in-phase and anti-phase modes between quasi-harmonic, yet goal-directed movements [5]. Details concerning this model, e.g. its stability properties can be found in [6].

#### Synchronization of single event pairs

In-phase and anti-phase synchronization between harmonic limit cycles is now generalized to synchronization modes of single event pairs in arbitrary combinations. Again, stable modes of synchronization are mapped to stable equilibrium points 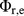 of the vector field 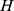. The values of 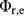, i.e. the locations in the attractor landscape, depend on the definition of the events 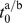 for which the initial phases (4) evaluate 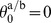. It makes sense to define them such that the pair 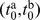 denotes a synchronization mode, with the corresponding attractor 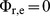. Using (11), the synchronization mode of any event pair 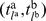 is then expressed by the equilibrium phase difference
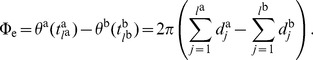
(19)


For each event pair representing a synchronized mode, the vector field 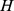 of the phase difference dynamics (14) needs to feature a point attractor 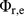, which is obtained from (19) with (10), see Fig. 5(b) for an example. The following points summarize the properties common to the design of the vector field 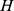:

- The phase plot is of oscillating shape, modeling an alternating sequence of attractors and repellors.
- The gradient and extrema in the vicinity of an equilibrium point 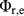 define its strength and region of attraction respectively [6], given a certain frequency detuning 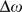.
- In order to obtain relative synchronization, we require 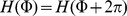.
- In contrast to the extended Kuramoto model and similar coordination models, symmetry 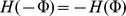 is generally not fulfilled.
- Positive (negative) values 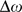 yield positive (negative) shifts of the attractor points.

*Note.* The attractor landscape of the phase dynamics becomes time-varying, if the relative primitive durations 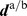 are subject to adjustment.

#### Synchronization of multiple events pairs

**Figure 6:**
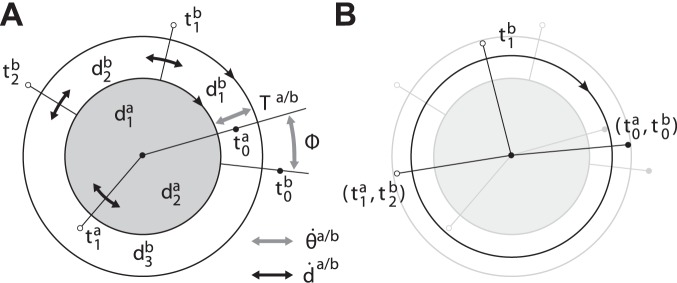
Circular illustration of the synchronization problem between two limit cycles. The exemplary limit cycles 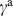 (inner circle) and 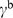 (outer circle) are introduced in Fig. 4. (A) The degrees of freedom available for synchronization: The periods 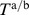 and the phase difference 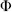 are both governed by the process (12), (13). The relative primitive durations 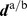 are governed by the process (20), (21). (B) Perfect synchronization of the event pairs 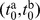 and 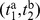, leading to coincident circles and events.

*Note.* Normalization is preserved e.g. by adjusting the components of 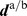 such that 
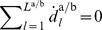
 holds, which is the derivative of the normalization constraint.

The coupled process (12), (13) accounts for synchronization modes that can be achieved by mutual entrainment of both periods and phase difference within certain domains of attraction. However, the simultaneous synchronization of multiple event pairs remains generally unexplained, as pointed out in the previous section. Therefore, the relative primitive durations 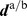 are proposed as additional degrees of freedom, governed by a cross-coupled dynamical process of the form
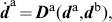
(20)




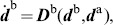
(21)with 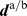 subject to normalization 
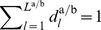
. In Fig. 6, the degrees of freedom of the overall synchronization process are illustrated for the above mode with respect to two event pairs. Synchronization modes that would require to accommodate large differences between components of 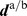 or between combinations thereof might be infeasible, e.g. due to velocity constraints related to the agents or their individual tasks. The process (20), (21) is therefore assumed to be subject to locally bounded regions of attraction. Such boundedness is similar to the range of frequency detuning 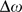 in (14), which limits stable phase synchronization.

In the following, we outline a possible realization of the process (20), (21) featuring the mode illustrated in Fig. 6(b). In this mode, the event pairs 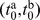 and 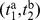 appear synchronized simultaneously. The former is readily synchronized by the phase dynamics (12), (13) employing the stable equilibrium point 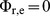. In order to additionally synchronize the latter, we design the entrainment of 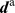 according to
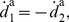
(22)




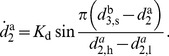
(23)


By (22), normalization is preserved. The gain 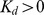 in (23) enforces the solution 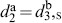 to be stable, saturated by
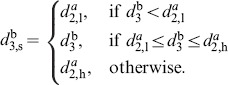



The thresholds 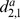 and 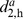 define the lower and upper bound on the entrainment of 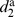. Assuming isotropic coupling between the agents, the entrainment of 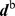 is designed analogously.

## Transformation between Movement and Phase

The synchronization process developed in the previous section governs the phase variables 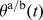 as well as the relative primitive durations 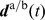. Since we target the integration of the synchronization behavior in the perception-action loop of robotic agents, the movement trajectories need to be transformed on-line into the process variables and vice versa.

### From Movement to Phase

The problem considered first is how to determine the partner’s phase *instantaneously*, based on measurements of the movement trajectory. Besides the instantaneous phase 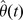, the solution presented in the following also provides event predictions 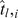, and thus via (2) and (3), predictions of the relative primitive durations 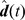.

#### Existing methods and open issues

Different methods have been applied to extract instantaneous phase variables from limit cycles that are known only by their observables, e.g. their cyclic movement trajectories. However, none of them fulfills our requirements entirely. First and foremost, only one-dimensional and narrow-band trajectories can be analyzed properly by the common methods. These methods are the analytic signal concept based on the Hilbert transform [42] and the state space methods [51] retrieving the trigonometrical phase angle in a position-velocity state space. Moreover, the former is restricted to off-line analysis, see [6] and [52] for a comparative discussion. The technique of linear phase interpolation between single marker events per period [42] can be considered analogous to the analysis of return times on the Poincaré map. Since this technique is applicable regardless of the frequency components and the dimensionality of the analyzed trajectory, we adopted our phase definition accordingly. However, the following challenges remain, preventing to calculate the phase straightforward via (4):

- Movement variabilities due to interaction or other perturbations considered as noise will cause the limit cycles of human partners to be *non-stationary*, i.e. the period 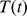 and thus, also the relative primitive durations 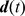 become instantaneous variables.
- The variables 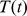 and 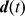 refer to a parameterization of the current period as a whole. Hence, on-line applications require estimates that are continuously predicting the future values these parameters take at period completion.

#### The instantaneous phase of non-stationary limit cycles

**Figure 7:**
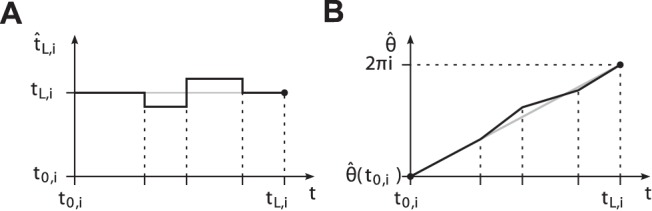
Instantaneous phase calculation. (A) Exemplary evolution of the predicted event 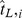 over time 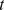. (B) Corresponding evolution of the phase 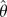 obtained from (25). The slope of 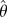 instantaneously relates the left over phase 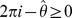 in period 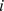 to the left over time span 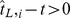. Black dots denote boundary conditions. Gray graphs depict perfect prediction and the harmonic phase respectively.

*Note.* Given a stationary limit cycle and assuming perfect prediction 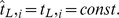, the solution of (24) can be derived analytically. It reads
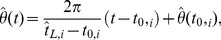
(26)which is obviously the harmonic angular phase complying with definition (4), cf. gray graphs in Fig. 7.

The desired phase variable is required to instantaneously reflect changes of the period, while it is also required to comply with the definition (4) prescribing the unperturbed phase evolution. Given a *prediction* of the event 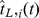 denoting the time of completion of the current period 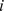, we propose a phase estimate for the time 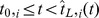 given by the solution of
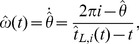
(24)which is a linear differential equation with time-varying coefficients. The initial condition reads 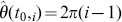. Time-varying predictions of the event 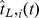 are instantaneously reflected by the phase velocity (24), see example plot in Fig. 7. Numerical integration of (24) yields the phase trajectory
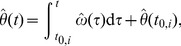
(25)which is due to 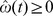 monotonically growing. For times 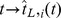, the solution of (24) converges to 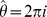.

#### Prediction of events from observation

Both the instantaneous phase (25) denoting the numerical solution of (24) and the relative primitive durations obtained from (2) and (3) require on-line predictions of the events 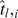, 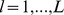 in the current period 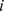. To that extent, we assume the state 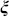 to be fully observable up to time 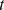. The task-related segmentation points 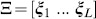 are assumed to be known and constant.

**Figure 8:**
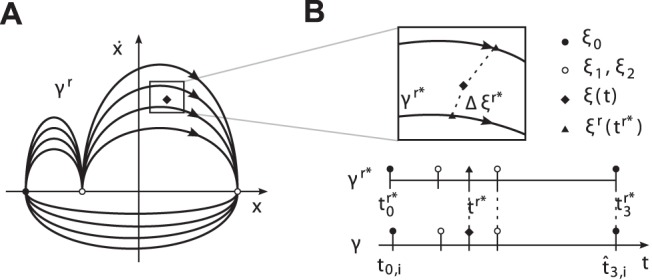
Classification-based event prediction. (A) Family of 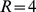 limit cycles 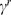 with differing periods 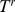. In the position-velocity state space, shapes differ due to 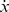 scaling with 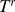. (B) Close-up illustrating distance-based classification *(top)*. Events are predicted based on the acquired evolution of events in 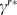 *(bottom)*.

We propose the following two-step technique to obtain predictions from experimental measurements:

- *Acquiring limit cycles:* Reference limit cycles
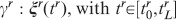
(27)are acquired over single, complete periods. A family of limit cycles 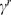, 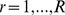 is built from a number of 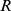 cycles. These feature differing periods 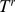 covering the expected range of periods, see example in Fig. 8(a).
- *Classifying limit cycles and predicting events:* The current state 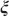 is classified with respect to the family of reference limit cycles. First, the similarity to each 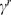 is determined by the respective minimum of the distance metric
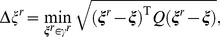
(28)with 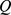 being a 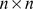 positive definite weighing matrix. Next, the closest cycle 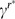 is selected by
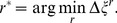
(29)

If the state 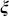 is close to the segmentation points, the distances 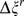 are nearly equal. In this case, undesired switchings of 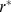 are avoided by switching from previous 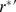 to current 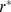 only if a certain threshold
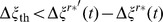
is exceeded. Finally, predictions of any future event 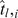 at time 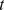 are obtained from
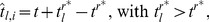
(30)where 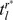 denotes the corresponding event in 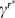 and 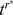 the time at minimum distance 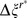 in 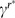, see Fig. 8(b).

*Note.* The quality of the event predictions depends on the number of reference limit cycles and their distribution of periods, i.e. how fine-grained the covered portion of the state space is sampled.

### From Phase to Movement

Robotic agents implementing the synchronization behavior require the transformation inverse to the previous one as well. By means of a technique based on movement models, the process variables are transformed back to the cyclic movement trajectory representing the individual action task. After defining the required model properties, we develop an exemplary realization of this transformation through a model based on the minimum-jerk criterion [53]. It renders human hand movements in goal-directed tasks [54].

#### General movement model

The trajectory is again composed by a given number of 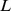 primitives 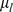, 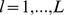 connecting the segmentation points 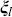 with relative primitive durations 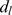. Inverse to the phase-amplitude decomposition of the cyclic state trajectory, we require the movement model to take the general form
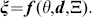
(31)


The function 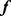 denotes a mapping of the phase 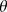, the distribution 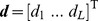, and the task-related segmentation points 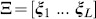 onto the continuous state trajectory 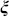. In brief, an appropriate movement model needs to.

- fulfill the condition (1) for finite periods 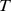,
- facilitate temporal scaling implemented by 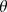 and 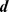,
- facilitate spatial scaling depending on 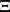.

Models complying with these properties are discussed in [27]. In the following, we re-parameterize a model 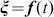 explicitly depending on time 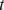 to comply with (31).

*Note.* The process variables 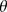 and 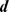 implement the degrees of freedom available for the *voluntary* behavior of movement synchronization. The movement model 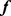 has to *necessarily* comply with the task-related segmentation points 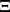.

#### The minimum-jerk model as an example

Human hand trajectories composed of point-to-point movements are known to be successfully reproduced by the minimum-jerk model formulated in a Cartesian frame [53]. With reference to the human-robot experiment described later on, we investigate this polynomial-type model. The state 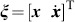 is defined, with 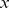 and 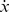 denoting the hand (effector) position and velocity in a Cartesian frame. The movement model (31) is then realized by a sequence of 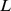 point-to-point primitives
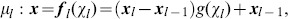
(32)parameterized by 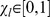. The function 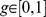 denotes the fifth-order polynomial



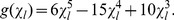
(33)The start point 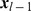 and the end point 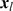 of the primitive 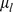 define the segmentation points 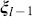 and 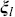, since (33) implies 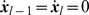. For any choice 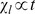, (32) minimizes the jerk 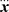.

#### Re-parameterization of the minimum-jerk model

The parameter 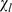 of the 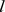
th primitive (32) is substituted by the process variables 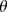 and 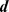, i.e.
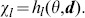
(34)


**Figure 9:**
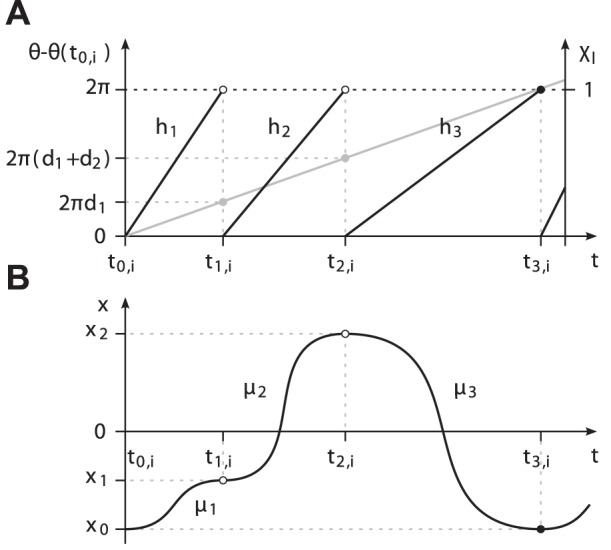
Transformation of the process variables 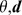 into a limit cycle with 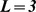 primitives 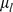. The minimum-jerk movement model is employed. (A) Piecewise-continuous substitutions 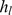 illustrated for the unperturbed phase with 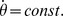 (gray graph) and 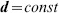. (B) Continuous, cyclic movement trajectory composed by polynomials 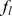. For the corresponding limit cycle representation, cf. 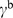 in Fig. 4A.

*Note.* If 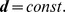 holds, 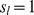 is satisfied, and the substitution (36) becomes piece-wise linear, i.e. 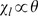. If additionally 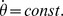 holds, piece-wise linear 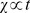 is obtained. Thus, if the synchronization process is in steady state, the trajectory 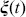 is composed by minimum-jerk movement primitives, cf. example in Fig. 9.

If (34) fulfills the condition
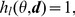
(35)the subsequent primitive is activated, i.e. the transition 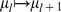 and 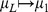 respectively is triggered, see Fig. 9(a). The substitution 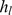 in the current period 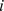 is realized by
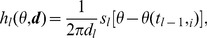
(36)which is composed as follows. The phase value 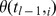 obtained from (11) is subtracted to remove the offset at the event of primitive entry 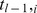. The factor 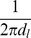 scales phase values 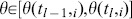 to values 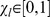. The term
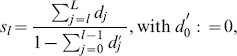
(37)ensures, that the boundary condition 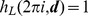 is fulfilled for any time-varying 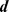. With 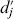 we denote the actual value that 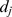 assumed at past transition 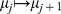.

## Human-Robot Synchronization Experiment

The concept of movement synchronization is applied to render the interactive behavior of a robotic agent that performs a joint action task together with a human partner. Supporting information is provided in Video S1. The human-robot synchronization experiment fulfills two goals. First, it provides a proof-of-concept implementation successfully illustrating the developed synchronization behavior by means of a robotic interaction partner. Second, it serves to explore the potentials of the developed robotic behavior in joint action tasks with human interaction partners.

In the following, superscripts ^a^ and ^b^ are replaced by ^H^ and ^R^ when variables belonging to the human and the robotic agent need to be distinguished.

### The Joint Action Task

The design of the experimental task is inspired by the dot-tapping paradigm studied in our previous work [5],[6]. The following points summarize the desired features:

- Both agents perform repetitive movements composed by sequences of *multiple primitives* with closed trajectories (cycles). Multiple cycles performed consecutively allow to study synchronization effects.
- Since we investigate different modes of synchronization, the cycles need to offer potentially relevant *synchronization events*.
- The task is *goal-directed*, i.e. the agents’ effectors have to reach one or more goal points.
- *Overlapping workspaces* provoke close interaction and constrain synchronization, since collision avoidance is required in certain workspace regions.
- Mutual pick up of *sensory information* about each others’ actions is allowed to let interaction emerge.

**Figure 10:**
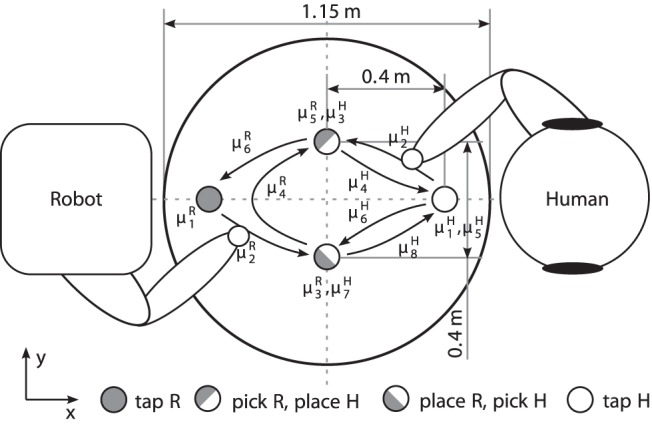
The joint action task designed for the human-robot synchronization experiment. In a symmetric setup, both human and robot perform slightly different action tasks while facing each other. Odd-indexed primitives 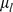 consider dwell times, even-indexed ones denote movements. Target points are marked by circles of 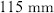 in diameter.

**Figure 11:**
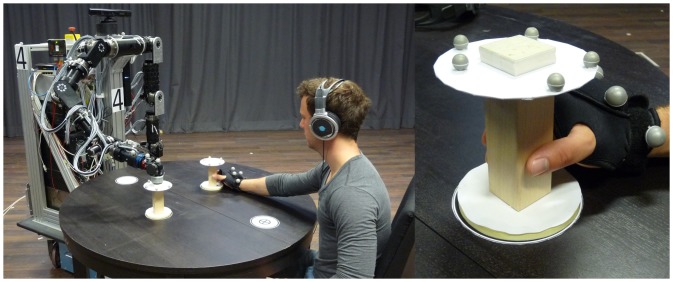
Experimental setup. *Left:* The scenario of a prototypical joint pick-and-place task. *Right:* Hand movements are made available to the robot in real time by tracking the glove the human interaction partner is wearing.

Accordingly, the task paradigm depicted in Fig. 10 is designed. Both the human and the robot perform cyclic sequences of multiple movement primitives with their right arm/manipulator, while sitting opposite to each other at a round table. The task is to carry barbell-shaped objects from pick points to place points which are marked on the table. The objects have a height of 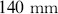 and a weight of 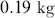; they are equipped with an iron sheet and a plastic disc on top with reflective markers attached, allowing for magnetic grasping and marker-based tracking respectively. The participant wears a glove with an additional weight and markers attached. Total weight of the glove is 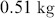. Its purpose is to naturally slow down the humans’ movements. The agents’ workspaces are arranged such, that two objects can be exchanged between them in a cyclic fashion. Within each pick-and-place movement, the table shall be touched at a tap point close to the agent. The robot only performs a tap when carrying an object, hence the agents’ movement cycles differ. A human-size mobile robot with anthropomorphic arms serves as the interaction partner in the experiment, see Fig. 11.

**Figure 12:**
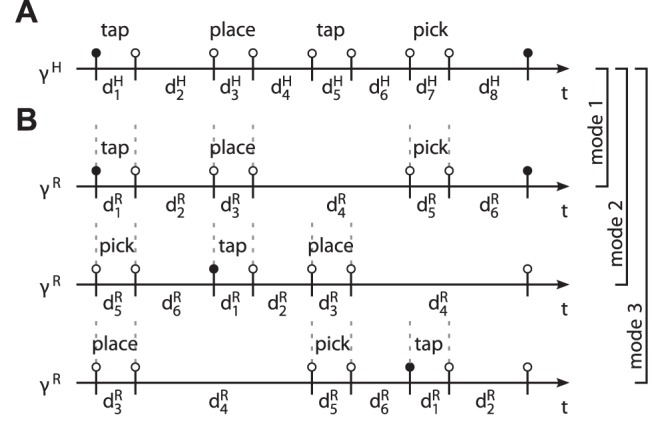
Evolution of events in the experimental task synchronized in three modes. The relative durations 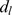 correspond to the primitives 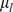 defined in Fig. 10. Again, odd-indexed durations are due to expected dwell times in the segmentation points. (A) The cycle  of the human. (B) The cycle 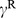 synchronized to 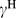 in three different modes, denoted mode 1–3. Vertical dashed lines indicate synchronized events. Intuitively speaking, the human precedes the robot in mode 2 and vice versa in mode 3.

Three synchronization modes are investigated in the above joint action task, see Fig. 12. These modes synchronize different combinations of pick, place and tap actions. Since the objects can be exchanged by sequential pick-and-place actions, the modes comply with the task-related goals. Note, that each of the segmentation points features two events, entry and leave of the respective point. These frame the so-called dwell time, which is known to be part of human motor control in aiming tasks [55]. The above modes are represented by stable equilibrium relations that are featured by the unified synchronization process, see Table 1. Details on the data acquisition system, the robotic system [56], [57] and the implementation of the synchronization behavior of the robot are given in the Appendix S1.

**Table 1:** Stable equilibrium relations of the synchronization process.

Mode	Phase difference Φ_r,e,*m*_	Relation of relative durations *d* ^H/R^
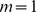	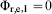	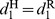 , 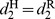 , 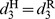 , 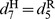 , 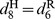
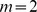	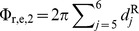	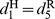 , 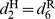 , 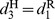 , 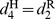 , 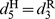
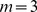	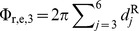	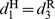 , 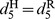 , 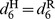 , 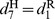 , 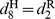

### Participants, conditions and procedure

#### Participants

Procedures were approved by the ethics committee of the medical faculty of the TUM and conformed to the principles expressed in the Declaration of Helsinki. In total, 12 people (9 female) participated in this experiment. They were between 20 and 48 years old (M = 30.8). All were right handed and had normal or corrected-to-normal vision and were nave as to the purpose of the experiment. For participation, they were paid 8 EUR per hour. Prior to their inclusion in the present study, all participants gave written informed consent. The individual in this manuscript has given written informed consent (as outlined in PLOS consent form) to publish these case details.

#### Conditions

Two conditions manipulated the synchronization behavior of the robot:

- **NOS**: **No S**ynchronization

The robot performed at 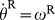, with constant frequency 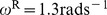. Its relative primitive durations were set constant to 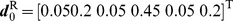.

- **PES**: **P**hase and **E**vent **S**ynchronization.

The robot aimed to synchronize the three modes we designed above, applying the parameters from NOS and the coupling gains 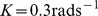 and 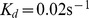.

In both conditions, the effector trajectory of the robot was subject to collision avoidance as described in the Appendix S1.

#### Procedure

The experimental procedure was as follows. The mobile platform of the robot was maneuvered to a target pose calibrated with respect to the table by means of markers, such that the goal points assigned to the robot were within the workspace of its right manipulator. Similarly, the participants were seated in a comfortable posture close to the table, cf. Fig. 10. A written instruction handed to the participants provided the description of the human-robot joint action task. In particular, the participants were advised that for the task to be successfully fulfilled, joint action in cooperation with the robotic partner is required. In order to provoke natural interaction, they were instructed to perform at comfortable speed and to touch the marked positions precisely in a single movement. Direct hand-over and sliding the objects over the table was not allowed. The participants were neither informed about the synchronization behavior of the robot, nor were they advised to synchronize. At the beginning of each trial, they were asked to rest with an object in their hand in the respective tap position and instructed to start executing the task as soon as they heard an acoustical start signal (high-pitched tone) through their head phones. The stop signal (low-pitched tone) was presented after they had performed ten cycles. The start signal was timed such that the modes described in Fig. 12 were provoked initially, i.e. for mode 1, both the participants and the robot were triggered simultaneously being in their tap points, for mode 2, the robot was triggered when the participants entered their place points, and for mode 3, the participants were triggered when the robot entered its place point. Six sets (two synchronization conditions × three start-off modes) each consisting of three trials were performed which led to a total of 18 trials. These sets were carried out in a randomized sequence of two blocks, each with three sets under the same synchronization condition. The sets manipulating the start-off mode were presented in randomized order in each block.

### Quantitative Measures

The following measures are deployed to assess the synchronization behavior observed in the experiment.

#### Event synchronization

The synchronization of events targeted by the behavioral model of the robot is assessed based on the measured Cartesian position trajectories of the human hand 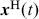 and the robot effector 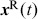. Those are recorded simultaneously by the motion capture system, thus differing processing delays are eliminated. Trajectory segmentation and event extraction is identical with the implementation of the robot. According to the definition of event synchronization above, we calculate for each synchronization mode 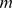 the temporal lags within all event pairs 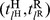 with the indexes 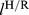 chosen corresponding to the events synchronized in mode 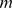. For each mode 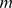, the lag magnitudes are averaged per period 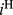, i.e. over event pairs with 
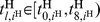
. Those averages provide continuous measures of asynchrony, which we denote ASYN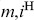. In each period 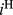, the best fitting one out of the three modes is detected by selecting the smallest asynchrony. The per-trial average of the latter over all periods 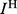 reads
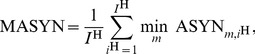
(38)which we call the mode-related asynchrony.

*Note:* The mode-related asynchrony quantifies the mean time lag between multiple event pairs measured in seconds. Only complete sets of event pairs corresponding to the defined modes are probed.

#### Mode distribution and mode switches

At any time, one of three synchronization modes is considered to be *active*, and pursued by the robot in condition PES. According to the vector field design cf. Appendix S1 and Table 1, we determine the active mode
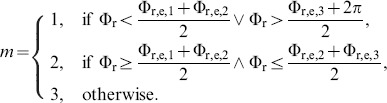
(39)


Given the evolution of the active mode 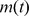, we analyze the relative distribution of modes 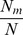 as an indicator of the within-dyad preferred synchronization mode, where 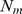 is the number of samples in active mode 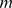 and 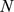 the total number of samples per trial. Note that the number of samples is representative of the continuous amount of time spent in a certain mode. Furthermore, the temporal persistence of modes is measured by the number of mode switches, i.e. the number of samples 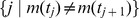 per trial.

#### Synchronization index

Phase synchronization is often quantified by means of the synchronization index, see e.g. [43] for a comprehensive review. Given the time series of the phase difference 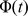 consisting of 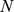 directional observations 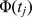, the synchronization index
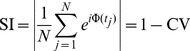
(40)is calculated, where CV denotes the circular variance of an angular distribution. The synchronization index SI is also called mean phase coherence. The synchronization concept in this article introduces multiple modes, represented by differing equilibrium phase differences. Trials with one or more mode switches would heavily degrade the index (40). Hence, we propose to calculate the synchronization index separately for epochs of the same *active mode*. The resulting indexes SI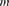 are then combined per trial into the *mode-related* synchronization index
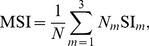
(41)weighted by the respective number of samples 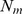.

*Note:* The 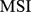 lies in the interval [0,1]. Given a perfectly uniform distribution of 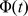, it would equal zero. It equals one only if the synchronization process is persistently in steady-state, which means that all samples of 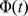 point to the same direction.

#### Entrainment error of relative primitive durations

As shown in our synchronization concept, the entrainment across the relative primitive durations 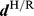 is essential to the synchronization of multiple event pairs. It is assessed by the root-mean-square error defined as the residual
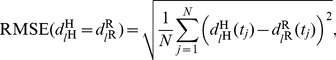
(42)with the primitive indexes 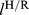 chosen corresponding to the equilibrium relations summarized in Table 1. For each relation and epoch of the same active mode 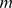, the entrainment errors are obtained from (42) and averaged over the five mode-dependent equilibrium relations afterwards, yielding the errors RMSE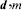. Analogously to the above definition of the mode-related synchronization index (41), those are then combined by the weighted average
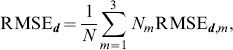
(43)which assesses the overall entrainment error of 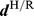.

## Experimental Results

The observable degree of event synchronization between the movements is evaluated as *external* measure. Feedback gathered from a short questionnaire is reported as well. We also assess the synchronization behavior through measures relying on *internal* variables of the robot. Note, that the results presented in the following are based on a group of nine participants unless stated otherwise. The remaining group of three participants performed at movement speeds either far below or above the speed range the robot is capable of moving at, thus impeding movement synchronization in the experiment. Possible reasons are discussed later.

### External Assessment of the Synchronization Behavior

The following results allow to explain, how far the overall goal of our synchronization concept is reached objectively, i.e. if it fosters the entrainment of movements by synchronizing multiple event pairs. In addition, subjective feedback from the participants gives rise to discuss some perceived effects.

#### Subjective reasoning

After having completed the experiment, participants were asked whether or not they had the feeling that the robot reacted to them. In case of a positive answer, they were asked to state if they found that perceived reactiveness pleasant (yes/no) and to reason about this answer. Eleven out of twelve participants recognized reactiveness of the robot in response to their movements during parts of the experiment. Ten out of eleven participants who answered positively stated that they liked the perceived reactiveness, giving reasons such as:

- It makes the robot appear lively.
- Having the control over task speed is pleasant.
- Adjustment towards similar speed is pleasant.
- It fosters smoother interaction.
- Negotiation among partners is beneficial.
- It is a nice feeling, but a bit uncanny as well.

The one who disliked the reactive behavior of the robot described the interaction as flurry and unsteady.

#### Event synchronization

**Figure 13:**
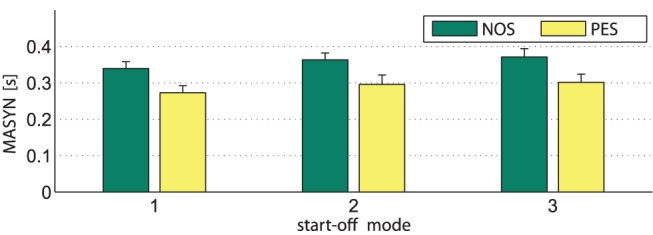
The mode-related asynchrony MASYN. Values are averages over all trials for the three start-off modes under the conditions NOS and PES. The bars represent standard errors of the mean.

The evaluation of the objective measure of event synchronization, which is the mode-related asynchrony MASYN, is depicted in Fig. 13. A 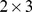 repeated measures ANOVA with the within subject factors *condition* (NOS, PES) and *start-off mode* (1–3) reveals a clear decrease of asynchrony in each of the start-off modes, 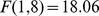, 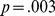, if the robot applies synchronization behavior, i.e. the condition PES. Irrespective of the synchronization condition, start-off mode 1 numerically results in lowest asynchrony values, whereas a slight trend towards increased asynchrony is visible for mode 2 and 3. However, differences between start-off modes were not significant and no significant interaction effect was observed, both 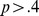.

### Internal Assessment of the Synchronization Behavior

In the following, the behavioral dynamics is evaluated based on its internal representation, i.e. the internal variables of the robotic agent.

#### Entrainment of phases and relative primitive durations

**Figure 14:**
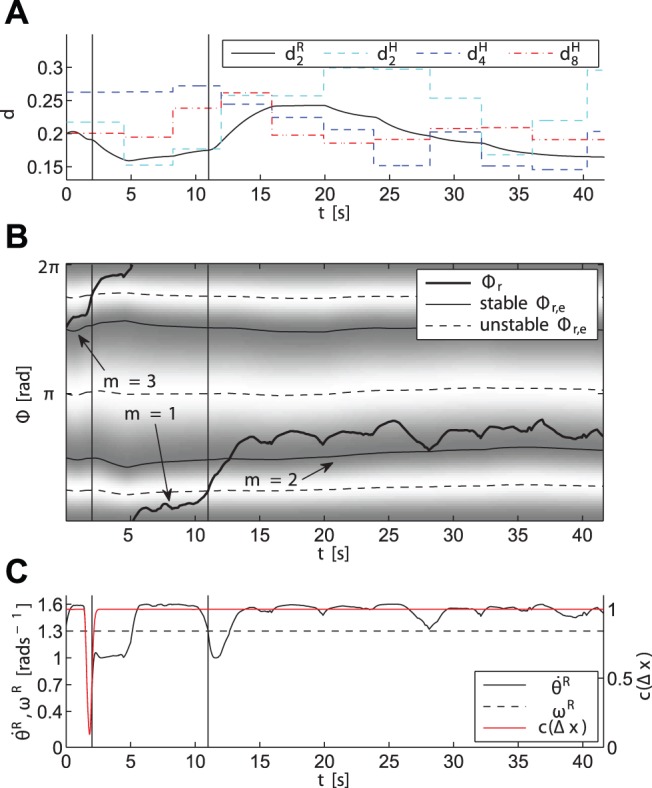
Evolution of selected process variables for a sample trial under condition PES and start-off mode 3. Vertical solid lines denote mode switches. (A) The duration 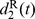 of the robot entrained with one of the durations 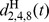 of the human, depending on the active mode. (B) The relative phase difference 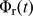, and the vector field 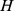 with its time-varying attractive regions (dark) and repulsive regions (bright) representing the modes 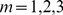. (C) The robot phase velocity 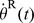 and collision avoidance function 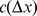.

To start, we explain the inner processes governing the synchronization behavior of the robot during an exemplary trial. The trajectories of relevant process variables are illustrated in Fig. 14. After starting off in mode 3, cf. initial phase difference in Fig. 14B, the relation 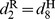 is entrained amongst others, see very left part in Fig. 14A. Note that the attractor landscape generated by the vector field 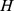 is morphed depending on the entrained components of 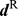. Thereafter, the phase velocity of the robot 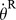 is slowed down by the function 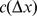 due to collision avoidance, Fig. 14C. As the participant progresses fast, the robot is forced into mode 1. Through modulation of 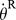 within the tuning range 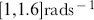, which is defined by its natural frequency 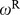 and coupling gain 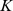, the robot attempts to sustain the mode it is close to. It can be seen, that now the relation 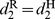 is pursued. After a while, the participant again increases speed, which leads the robot to finally switch to mode 2. Here, the relation 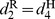 becomes entrained.

#### Preferably synchronized modes

**Figure 15:**
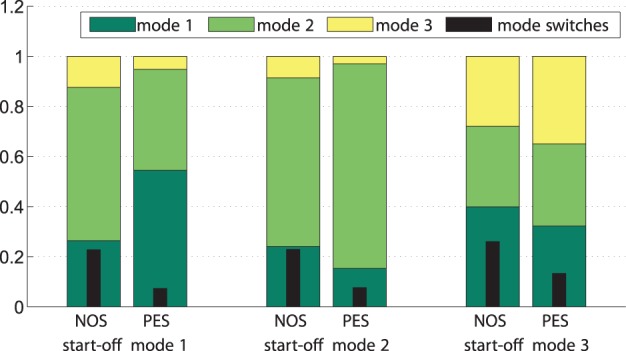
Relative amount of time spent in each mode and relative amount of mode switches. Both are averaged separately over all trials for the three start-off modes under the conditions NOS and PES.

The relative amount of time spent in the synchronization modes and the relative amount of mode switches are illustrated in Fig. 15. Here, the relative time spent in each mode provides an intuition of how long, on average and with respect to the trial durations, each mode has been active within the robot behavior. It can be seen that under PES, the relative share of that mode increases, which the human-robot dyad has started with (upon trigger). To access the differences between NOS and PES with regard to the amount of time spent in triggered mode, planned comparisons were performed between conditions (NOS, PES) within the respective start-off mode. If participants were triggered to start off in mode 1, the relative amount spent in mode 1 is significantly higher under PES compared to NOS, 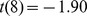, 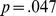. Since under NOS, the robot only observes but not actively pursues these modes, that increase is due to robotic synchronization behavior in PES. Similar results were obtained for start-off mode 3, 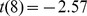, 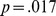. However, the difference between relative mode share in PES and NOS during start-off mode 2 was only found to be numerical, 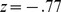, 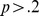. Mode 2 was also the dominant mode during NOS. Hence, no effect of the synchronization behavior is visible here. Overall this shows that when being triggered close to the attracted modes, the robot successfully sustains them.

This is also reflected by the relative share of mode switches. Results of a 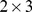 repeated measures ANOVA on condition and start-off mode show that the amount of mode switches decreased under PES in each of the start-off modes 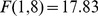, 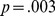. Neither a difference between start-off modes nor an interaction effect was observed, 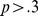.

**Figure 16:**
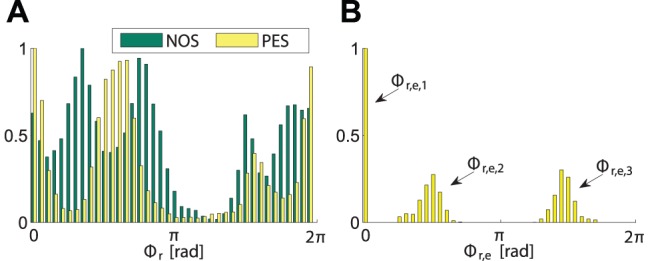
Relative frequencies of occurrence of phase differences. (A) The relative phase difference 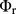 under the conditions NOS and PES. (B) The attracted equilibrium phase differences 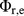 under PES.

The preferred phase relations as a result of phase synchronization are reflected by histograms of the phase difference, see Fig. 16 left, which is a representation complementary to the mode distributions above. Some preference towards certain phase relations can be recognized even under condition NOS, which is ascribed to human synchronization attempts due to the static behavior of the robot. Under PES, the distribution gets sharpened, forming three distinct peaks. When comparing that distribution in Fig. 16 left with the distribution of actively attracted equilibrium points in Fig. 16 right, their coincidence indicates successful phase entrainment through the robot behavior. Weight on the peak corresponding to mode 2 (i.e. 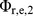) is strongest, followed by the peak at mode 1 (i.e. 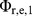), which is in line with the distribution of modes in Fig. 15. Note that the smeared distributions of 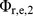 and 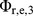 are due to their dependency on the relative primitive durations 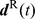.

#### Quantitative assessment of the synchronization process

**Figure 17:**
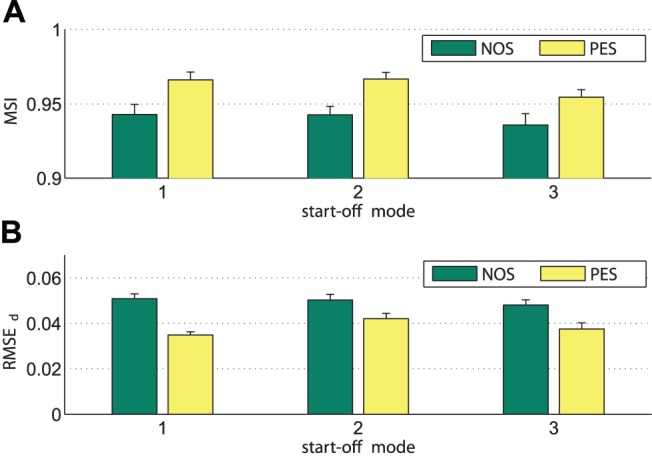
Entrainment measures. Values are averages over all trials for the three start-off modes under the conditions NOS and PES. The bars represent standard errors of the means. (A) The mode-related synchronization index MSI. (B) The root-mean-square error of durations entrainment RMSE***_d_***.

The convergence and performance of the dynamical process of synchronization is measured by means of the process variables, which are the phases or the phase difference 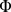 respectively, and the relative primitive durations 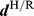. The results are illustrated in Fig. 17. To access the differences between NOS and PES governed behavior, 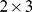 repeated measures ANOVAs were performed with the within subject factors condition and start-off mode. For MSI, the condition PES causes an increased entrainment compared to NOS, 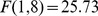, 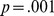, see Fig. 17A. Between start-off modes no significant difference was observed, 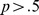. Also no significant interaction effect was detected. Similar results are obtained for the entrainment errors of durations, which are decreased by the entrainment process under PES, 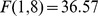, 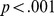, see Fig. 17B. Lowest errors with respect to the attracted equilibrium relations are achieved in start-off mode 1 under PES, as shown by a significant interaction effect, 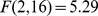, 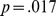.

#### Instantaneous phase estimation

**Figure 18:**
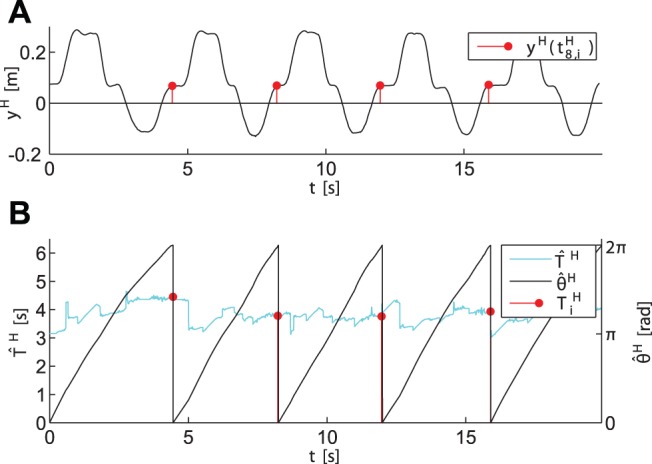
Evolution of the instantaneous phase estimation for the first half of the sample trial. (A) The 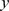-component of the human hand position, and the events 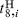. (B) The estimated instantaneous period 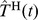, the measured period 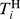, and the estimated phase 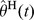 taken modulo 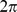.

The characteristic evolution of the period and phase estimation obtained from the human movements are illustrated by means of the sample trajectories depicted in Fig. 18. The events 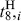 result from on-line segmentation of the movement trajectory 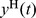, see Fig. 18A. Those events denote the time of the human hand entering the tap point, and the completion times of the periods 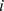. The instantaneous period 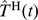 depicted in Fig. 18B is equivalent to the prediction 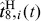, due to the definition of the instantaneous period 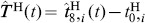. For comparison, the values 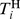 measured at period completion are shown as well. Note that due to the finite number of reference cycles used for event prediction, 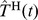 is not continuous. More specifically, when the reference cycle selected by classification switches, corresponding event predictions switch as well. It can be seen that the on-line estimation of the human phase 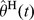 successfully satisfies our demands: It reflects changes of 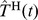 instantaneously and smoothly, while it still remains 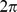-periodic with respect to the events 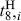 marking the period completions.

## Discussion

The results gained from the human-robot synchronization experiment provide the proof of concept and evidence the potentials of synchronization behavior in human-robot joint action. In brief, the following novel insights beyond existing research on movement synchronization are identified: New synchronization modes are explored in the context of goal-directed joint action tasks. The mode-related asynchrony MASYN is successfully decreased by the proposed unified entrainment process of both phase and relative primitive durations. Therefore, the interactive behavior of the robot driven by the proposed concept under the condition PES significantly improves the overall degree of synchronization between the robot and human partners compared to the static behavior under NOS. Mixed discrete and continuous repetitive movement primitives are synchronized in the pick-and-place task. Hence, the novel phase estimation technique is evidently applicable to multi-primitive movement cycles, which cover a wide range of repetitive joint action tasks. Subjective feedback reveals that the synchronization attempts of the robot towards the designed modes lead to an enriched sense of interaction with the robot for most of the participants. This highlights the strong potential of this approach to advance the social interaction capabilities of robots that perform joint actions with humans. In the following, the synchronization concept and the experimental results are discussed in more detail, both in the light of human-robot joint action and the design of interactive behavior for artificial agents.

### Implications on Human-robot Joint Action

Both the objective improvement of event synchronization achieved in our exploratory study and the summary of subjective feedback underpin the endeavor to investigate synchronization behavior evident in human-human interaction in the context of human-robot joint action. The behavioral dynamics pursues weak phase synchronization enforced by sinusoidal coupling of strength 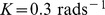, which is close to the coupling strengths of uninstructed human-human movement synchronization identified in [6]. Thus, the applied weak forcing is such that participants could not only switch between synchronization modes, they also could have easily resisted or distorted synchronization within the constraints imposed by the hand-overs. In support of this, for a group of three participants we observed that, under PES, the degree of synchronization deteriorated which stands in contrast to the reported improvement of synchronization for the group of nine participants. Most participants appreciated the synchronization attempts of the robot. In short, their answers let us conclude that they had an enriched sense of interaction.

However, the objective results also suggest some implications and pitfalls that need to be addressed carefully in the design of synchronization behavior. Subjectively pleasant, mutual entrainment of movements appears to be rather sensitive to parameterizations of the behavioral dynamics, first and foremost their attractors and their associated strengths. If those do not match the individual entrainment behavior of the human counterpart within certain ranges, inter-agent entrainment may fail and even worse: artificial entrainment attempts may be misinterpreted and lead to a degraded sensation of interaction compared to non-reactive behavior.

The appearance of the robotic partner and, strongly connected, its capabilities anticipated by the participants, is expected to also affect human interactive behavior [58]. Besides its manipulator kinematics having similarity to the human arm, the design of the robotic agent used in our study is rather associated with functional and technical attributes, than with humanoid ones, see Fig. 11. Moreover, we did not brief the participants on the behavior they could expect from their robotic partner. One of the participants reported to perceive some uncanny-ness when facing the reactive behavior of the robot, which could likely be originated by the potential mismatch of rather crude appearance and sophisticated interaction capabilities. Both factors give rise to further investigations going beyond the scope of this study.

Human interactive behavior may furthermore heavily depend on how the task context is conveyed and understood [59]. Human-robot experiments are usually conducted within controlled laboratory settings, which makes it hard to reliably create the desired context in the participants’ minds, e.g. that of an everyday activity performed within familiar surroundings. While the implemented behavior is geared towards the abstract context of joint action, emphasis on the individual performance requirements and the cooperative aspect of the task is likely to vary between participants. For example, the instruction to precisely hit designated tap points might be assigned higher priority than an uninstructed and likely unconscious desire to reduce dwell times of the partner in favor of smooth and fluent interaction. All of the above discussed points may affect uninstructed, i.e. emerging synchronization behavior in human-robot joint action. We hypothesize that among those points reasons can be found for the hampered synchronization and behavioral mismatch we observed within the disregarded group of three participants.

### Design Issues Regarding the Synchronization Concept

One key idea of the synchronization concept is the design of synchronization modes by means of a dynamical synchronization process unifying both phase synchronization and the entrainment of relative primitive durations. It has to be emphasized that both processes are usually cross-coupled: The entrained components of 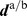 depend on the attracted mode 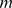, cf. Table 1 defining the modes implemented in the experiment. Changes of the relative primitive durations 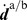 due to mutual entrainment cause shifts of the equilibrium points 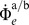 within the phase dynamics on the one hand. On the other hand, the attracted mode 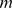 is determined by the equilibrium point which is closest to the phase difference 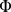. Depending on the designed modes and their attractor dynamics, the interaction of both processes might not be generally stable by itself, and therefore eventually result in oscillations between attracted modes. By defining reasonable bounds 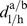 and choosing the gain 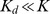, the entrainment process of durations is bound to certain attractor regions and slow compared to the phase difference dynamics. Though we did not encounter that kind of instability in our experimental setting, the formal derivation of stability bounds remains an open issue.

The presented design of synchronization behavior offers several interesting degrees of freedom which are not investigated in this article. The structure of the phase synchronization process is originated from the extended Kuramoto model [6] and variants of the HKB model [14] respectively, which evidently replicate human synchronization behavior. In contrast, the implemented entrainment structure of the relative primitive durations is considered prototypical, leaving room for further investigation and validation in the field of human-robot joint action. Similar to the phase dynamics of the HKB model, synchronization modes can be assigned differing weights through variable strengths of attraction. Another degree of freedom is provided by the natural frequency. In line with oscillator theory, the natural frequencies of the harmonic oscillators govern the individual behavior, since they autonomously drive the agents’ task progress at their individually desired speed. The domains of successfully negotiated entrainment between the agents is defined by the frequency difference.

Since the definition of the instantaneous phase purely depends on recurrent events within the period, the movement trajectory can be of arbitrary shape, as long as predictions of those events are provided. Instead of the presented technique based on minimum distance classification in the state space, the application of machine learning techniques such as programming by demonstration [60] could be investigated alternatively, for the sake of a flexible encoding of observed movement sequences and event predictions.

## Summary and Conclusion

In this article, we propose a novel concept and design methodology to synthesize goal-directed synchronization behavior for robotic agents in repetitive joint action tasks. Those tasks are assumed to be performed by dyads of agents in a common interactive setting. We only require the tasks to be described by closed trajectories in state spaces, where the states capture the relevant movements. Based on oscillator theory, the closed state trajectories are interpreted as limit cycles, for which corresponding phase variables are derived. The sought phases reflect the expected non-stationarity in the limit cycles instantaneously, or in other words, they are defined on a within-period scope and determined on-line. Goal-directed repetitive movements are shown to contain much richer information concerning synchronization than purely their oscillating property captured by the phase variable. Through segmentation, we split complex movement trajectories into sequences of multiple primitives, which are separated by events, e.g. the occurrence of points with zero velocity. Beyond in-phase and anti-phase known from harmonic oscillations, enhanced synchronization modes within limit cycle pairs are synthesized. Their definition utilizes both continuous phases and discrete events as anchoring points for synchronization. The key idea of the synchronization concept is the design of interactive behavior synchronizing the synthesized modes by dynamical processes. In a unifying view, the entrainment of both phases and primitive durations is designed to happen simultaneously on a continuous time scale, as mutual state feedback is assumed to be continuously available to the agents. Inverse to the phase estimation problem, action taking of the robotic agent governed by the synchronization behavior is addressed as well. In the prototypical scenario of a repetitive pick-and-place task, we enable a full-sized, anthropomorphic robot driven by the synchronization concept to cooperate with a human partner. Both objective synchronization measures and subjective feedback evidence effectiveness of the synchronization behavior. Besides the proof of concept, the results gained from the exploratory study highlight the potential of the synchronization concept to enhance the social competence of robots interacting with humans.

The continuous attractor dynamics of the synchronization behavior facilitates the intuitive and systematic design of goal-directed movement coordination. Therefore, the synchronization concept is considered as a promising enhancement to the approach of central pattern generators in the field of robotics. Applications ranging from intra- to inter-agent action coordination are worth looking at in this line of research. We expect the risk of mutual entrainment mismatch in human-robot interaction to diminish, if the behavioral rules of entrainment are derived from observations of human-human interaction. Furthermore, humanoid robots as interaction partners should be employed in realistic joint action scenarios, in order to ultimately disentangle the effects of robotic motor coordination on human-robot joint action.

## Supporting Information

Video S1 **Details of the experimental scenario and the implementation.**
(MP4)

Appendix S1 **Experimental setup and implementation.**
(PDF)
