# Supplementary material for: Rhythm Patterns Interaction - Synchronization Behavior for Human-Robot Joint Action
Source: PLoS One. 2014 Apr 21;9(4):e95195. doi: 10.1371/journal.pone.0095195 (PMC3994045; doi:10.1371/journal.pone.0095195)
Supplement: Appendix S1 — Experimental setup and implementation. (PDF) [file pone.0095195.s002.pdf]

Appendix S1 - Experimental setup and implementation

Experimental setup

Motion capture system

A marker-based motion capture system *(Qualisys)* is employed to visually track the Cartesian positions of the moving bodies, i.e. the human hand, the robot effector and the objects. The motion capture system consists of ten networked cameras *(Qualisys Oqus 4)*, that are mounted approximately 3 m above the work space and distributed on a square of approximately 10 m × 10 m. Prior to the experiment, the system is calibrated within the work space by means of a manual calibration routine, which guarantees an accuracy < 3 mm. A set of four passive markers is rigidly attached to each body to be tracked in a unique geometrical configuration for proper identification. Ball-shaped, reflective markers of 12 mm and 20 mm diameter are used. The software suite *Qualisys Track Manager (QTM)* for interaction with the system runs on a *Microsoft Windows*-based personal computer. It provides the capture data of the three-dimensional body coordinates with respect to the Cartesian frame attached to the table center. Via the real-time interface, capture data are made available to the robot on-line at a frequency of 200 Hz and at low latencies through a network connection. Low-pass filtering is applied to the data using a 25-point moving average FIR filter at a sampling rate of 1 kHz.

Robotic agent

The human-sized mobile robot is equipped with a pair of seven degrees-of-freedom manipulators [56] of anthropomorphic dimensions. An admittance-type control scheme based on a wrench sensor *(JR3)* in the wrist of the robot realizes compliant behavior of the manipulator when touching the environment. The effector of the right manipulator is equipped with an electromagnetic gripper which allows fast grasps and releases of ferromagnetic objects. A marker-to-effector calibration routine enables robust vision-guided grasping of marked objects by minimizing the error between marker positions and the effector position the manipulator is controlled to. Details on the software architecture can be found in [57]. The algorithms implementing the estimation of the human phase, the synchronization processes, the trajectory generation and the manipulator control scheme are developed in *MATLAB/Simulink*. Utilizing *MATLAB Real-Time Workshop*, the corresponding routines are executed at a sampling rate of 1 kHz on the on-board PCs of the robot running *Ubuntu Linux*. The overall processing delay between perception and action is approximately $\Delta t_{\mathrm{p}} = 30\,\mathrm{ms}$, which is the average time elapsing from marker movement until movement response of the robot.

Implementation

Design of the synchronization behavior

The vector field H is designed, which defines the phase difference dynamics $\dot{\Phi} = \Delta\omega + H(\Phi)$, with the phase difference $\Phi = \theta^{\mathrm{H}} - \theta^{\mathrm{R}}$. The unstable equilibrium points separating the regions of attraction are equally spaced between the stable equilibrium points given in Table 1, see Fig. S1. By splitting the phase difference dynamics under the assumption of isotropic coupling, we obtain the cross-coupled phase entrainment process

$$\begin{aligned}
\dot{\theta}^{\mathrm{H}} &= \omega^{\mathrm{H}} + \frac{H(\theta^{\mathrm{H}} - \theta^{\mathrm{R}})}{2} \\
\dot{\theta}^{\mathrm{R}} &= \omega^{\mathrm{R}} - \frac{H(\theta^{\mathrm{H}} - \theta^{\mathrm{R}})}{2}.
\end{aligned}$$

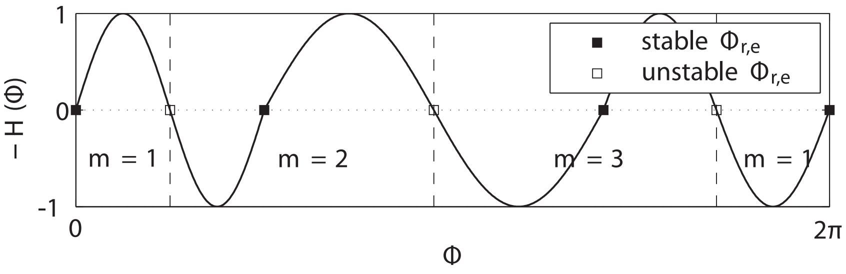

Figure S1. The vector field function H designed by continuous concatenation of sine periods. The stable equilibrium points are chosen according to Table 1. Vertical dashed lines separate the regions of attraction for the case of equal frequencies $\Delta\omega = 0$ and define the active mode m. The plot is parameterized by unit amplitude and $\boldsymbol{d}^{\mathrm{R}} = [0.05\ 0.25\ 0.05\ 0.4\ 0.05\ 0.2]^{\mathrm{T}}$.

The processing delay Δt_{p} of the robot is compensated by adding the constant phase shift $\Delta\hat{\theta}^{\mathrm{H}} = \omega^{\mathrm{R}}\Delta t_{\mathrm{p}}$ to the human phase estimate $\hat{\theta}^{\mathrm{H}}$.

The entrainment process of the relative primitive durations is realized according to the above developed example, i.e. according to (22) and (23). Within the regions of attraction defined by the lower bounds $\boldsymbol{d}_{\mathrm{l}}^{\mathrm{R}} = \frac{1}{2}\boldsymbol{d}_0^{\mathrm{R}}$ and the upper bounds $\boldsymbol{d}_{\mathrm{h}}^{\mathrm{R}} = \frac{3}{2}\boldsymbol{d}_0^{\mathrm{R}}$ around the initial values $\boldsymbol{d}_0^{\mathrm{R}}$ and depending on the active mode m, the equilibrium relations summarized in Table 1 are attracted.

Transformation between movement and phase

The instantaneous phase estimate $\hat{\theta}^{\mathrm{H}}(t)$ is determined according to the classification and prediction technique proposed above. The state $\boldsymbol{\xi}^{\mathrm{H}} = [y^{\mathrm{H}}\ \dot{y}^{\mathrm{H}}]^{\mathrm{T}}$ is defined, with y^{H} and $\dot{y}^{\mathrm{H}}$ denoting the y-components of the tracked Cartesian position and velocity of the human hand. Velocity is obtained from numerical differentiation. For on-line segmentation, the threshold velocity $|\dot{y}^{\mathrm{H}}| = 0.03\,\mathrm{ms}^{-1}$ is used. Event prediction for phase estimation is performed based on $R = 21$ reference limit cycles that have been generated by the minimum-jerk movement model, see Fig. S2. The weighing of position and velocity is defined by the matrix $Q = \mathrm{diag}(1, 0.7)$. The metric difference threshold is set to $\Delta\xi_{\mathrm{th}} = 0.05$. The relative primitive durations are sampled at completion of each cycle i, i.e. $\boldsymbol{d}^{\mathrm{H}}(t_{8,i})$, through on-line segmentation of the human trajectory and averaged over the last three values.

The effector trajectory of the robot is realized by the minimum-jerk model described above, which yields the fixed path depicted in Fig. S3. The pick positions of the objects are visually tracked during interaction, whereas the place positions are calibrated in advance via markers.

Collision avoidance

Whenever the effector is close to either the human hand or to an empty pick/occupied place position, the phase velocity of the robot is modulated by

$$\dot{\theta}^{\mathrm{R}\prime} = c(\Delta x)\dot{\theta}^{\mathrm{R}}.$$

Depending on the Euclidean distance Δx between the effector position and the human hand or the occupied/empty goal points, the smooth blending function

$$c(\Delta x) = \begin{cases} 0, & \text{if } \Delta x < \Delta x_{\mathrm{l}}, \\ \frac{1}{2} - \frac{1}{2}\cos\left(\pi\frac{\Delta x - \Delta x_{\mathrm{l}}}{\Delta x_{\mathrm{h}} - \Delta x_{\mathrm{l}}}\right), & \text{if } \Delta x_{\mathrm{l}} \leq \Delta x < \Delta x_{\mathrm{h}}, \\ 1, & \text{otherwise} \end{cases}$$

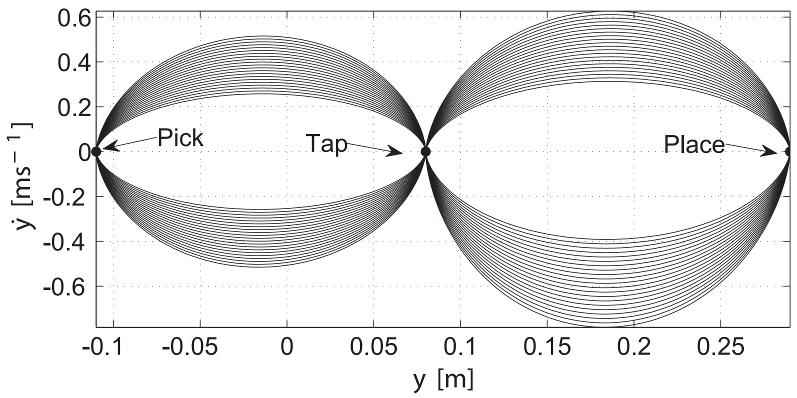

Figure S2. Phase plot of the family of reference limit cycles. Phase velocities $\dot{\theta} \in [1, 2]$ rads^{-1} are chosen equally distributed. Positions are expressed in the table-centered frame, which is aligned to the frame in Fig. 10. The primitive durations are set to $\boldsymbol{d}_0 = [0.05\ 0.2\ 0.05\ 0.16\ 0.05\ 0.22\ 0.05\ 0.22]^{\mathrm{T}}$. Those and the segmentation points denoted by filled dots are mean values which resemble the observations made from pilot trials with a human experimenter.

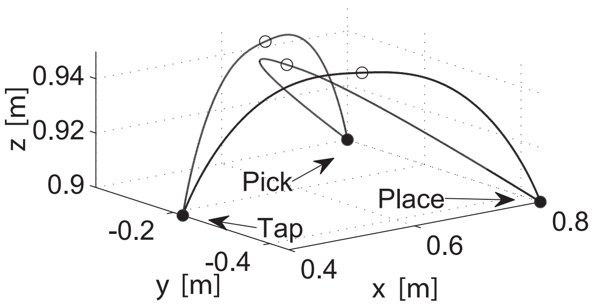

Figure S3. Cyclic effector path of the robot obtained from the minimum-jerk model, expressed in robot coordinates. Filled dots denote segmentation points, open dots denote via points with relative elevation of 0.05 m above the xy-plane.

is applied, which implements a simple collision avoidance behavior. Within the upper distance bound set to $\Delta x_{\mathrm{h}} = 0.25\,\mathrm{m}$, the phase velocity $\dot{\theta}^{\mathrm{R}'}$ is gradually slowed down to zero, reached at the lower distance bound $\Delta x_{\mathrm{l}} = 0.15\,\mathrm{m}$.
